# Weight training and risk of all-cause, cardiovascular disease and cancer mortality among older adults

**DOI:** 10.1093/ije/dyae074

**Published:** 2024-06-03

**Authors:** Prathiyankara Shailendra, Katherine L Baldock, Lok Sze Katrina Li, Jessica Gorzelitz, Charles E Matthews, Britton Trabert, Jason A Bennie, Terry Boyle

**Affiliations:** Australian Centre for Precision Health (ACPreH), University of South Australia, Adelaide, SA, Australia; Allied Health and Human Performance, University of South Australia, Adelaide, SA, Australia; Australian Centre for Precision Health (ACPreH), University of South Australia, Adelaide, SA, Australia; Allied Health and Human Performance, University of South Australia, Adelaide, SA, Australia; Allied Health and Human Performance, University of South Australia, Adelaide, SA, Australia; Department of Health and Human Physiology, University of Iowa, Iowa City, IA, USA; Metabolic Epidemiology Branch, Division of Cancer Epidemiology and Genetics, National Cancer Institute, Bethesda, MD, USA; Metabolic Epidemiology Branch, Division of Cancer Epidemiology and Genetics, National Cancer Institute, Bethesda, MD, USA; Metabolic Epidemiology Branch, Division of Cancer Epidemiology and Genetics, National Cancer Institute, Bethesda, MD, USA; Obstetrics and Gynecology, University of Utah, Huntsman Cancer Institute at the University of Utah, Salt Lake City, UT, USA; Population Health Unit, Murrumbidgee Primary Health Network, Wagga Wagga, NSW, Australia; Australian Centre for Precision Health (ACPreH), University of South Australia, Adelaide, SA, Australia; Allied Health and Human Performance, University of South Australia, Adelaide, SA, Australia

**Keywords:** Mortality, muscle-strengthening activity, older adults, physical activity, strength training, resistance training

## Abstract

**Background:**

While previous studies indicate muscle-strengthening exercises may reduce mortality risk, further research is needed to increase certainty of the evidence. We investigated overall and dose-response associations between weight training and the risks of all-cause, cardiovascular disease (CVD) and cancer mortality in a large cohort of older adults with long follow-up time and a large number of deaths. We also investigated the joint associations of weight training and aerobic exercise with mortality risk.

**Methods:**

Weight training was assessed via self-report in 2004–05 in the National Institutes of Health-American Association of Retired Persons (NIH-AARP) Diet and Health Study (USA; *n* = 216 339), with follow-up to 2019. Cox regression estimated the hazard ratios (HR) and 95% confidence intervals (CI) for the associations between weight training and mortality, after adjusting for confounders including aerobic exercise.

**Results:**

Around 25% of participants [mean age = 69.9 years (standard deviation = 5.4), 58% men] reported engaging in weight training over the past year, and there were 79 107 (37%) deaths. Engaging in any weight training (vs none) was associated with lower risks of all-cause (HR = 0.94; 95% CI = 0.93–0.96), CVD (HR = 0.92; 95% CI = 0.90–0.95) and cancer mortality (HR = 0.95; 95% CI = 0.92–0.98). More time spent in weight training was associated with only marginally greater risk reductions. Larger risk reductions were observed among women than men. Performing both aerobic exercise and weight training conferred the greatest mortality risk reduction; weight training was not associated with mortality risk among participants who did no aerobic exercise.

**Conclusion:**

Performing any amount of weight training lowered mortality risk.

Key Messages

- Performing any amount of weight training was associated with lower risk of all-cause, CVD and cancer mortality in this cohort of older adults, and those who engaged in both weight training and aerobic exercise were at lowest mortality risk.
- Larger risk reductions were observed among women than men.
- Further emphasis should be placed on the benefits of muscle-strengthening activities for overall health and mortality reduction in public health programmes.

## Introduction

National and global physical activity guidelines recommend that, in addition to performing moderate-to-vigorous intensity aerobic physical activity (MVPA; e.g. brisk walking, running, cycling), adults should engage in muscle-strengthening exercises involving all major muscle groups on 2 or more days per week.^1–3^ Muscle-strengthening exercises, sometimes referred to as resistance training or strength training, involve using weight machines, resistance bands, free weights or body weight to strengthen the musculoskeletal system.^4^

Research indicates engaging in muscle-strengthening exercises has a range of cardiometabolic, musculoskeletal and mental health benefits,^5–9^ and that the combination of both muscle-strengthening exercises and aerobic exercise leads to greater improvements in anthropometric outcomes, metabolic syndrome factors and cardiovascular risk markers than performing either of those activities alone.^10^^,^^11^ Findings from cohort studies suggest muscle-strengthening exercises may be associated with a lower risk of developing type 2 diabetes, cardiovascular disease (CVD), obesity and some cancers.^12–14^ However, although numerous studies suggest aerobic exercise is associated with lower mortality risk among the general population and older adults,^3^^,^^15^ there are considerably fewer studies on muscle-strengthening exercises and mortality risk.

The research to date has generally found that muscle-strengthening exercises reduce the risk of all-cause mortality.^14^^,^^16^ A 2022 systematic review and meta-analysis of 16 studies, seven of which examined all-cause mortality, found engaging in muscle-strengthening exercises reduced all-cause mortality risk by 15% compared with doing none.^14^ Subsequent studies have observed risk reductions of similar magnitude.^17^^,^^18^ Engaging in muscle-strengthening exercises has also been associated with a reduced risk of CVD mortality and cancer mortality; however, there are fewer studies and the results are inconsistent.^13^^,^^16^ Previous studies have generally had information about frequency (e.g. sessions/week) rather than time spent performing muscle-strengthening exercises (e.g. min/week), so the impact of higher volumes of muscle-strengthening exercises on mortality is uncertain.^14^^,^^16^ Previous studies have also had relatively small sample sizes, small numbers of deaths and/or short follow-up times, which has limited their precision when estimating the association between muscle-strengthening exercises and mortality risk. As such, several areas require further investigation. These include whether the association differs by sex, and whether performing both muscle-strengthening and aerobic exercise (as recommended by physical activity guidelines) confers additional mortality benefits compared with either activity alone.^14^^,^^16^ Finally, it is unclear if muscle-strengthening exercises confer mortality benefits among older adults, as most studies to date have been conducted in study populations with adults of all ages.

The aim of this study was to investigate the dose-response associations between weight training—a specific type of muscle-strengthening exercise—and all-cause mortality, CVD mortality, and cancer mortality overall, stratified by sex in a large cohort of older adults with long follow-up time and a large number of deaths. We hypothesized that weight training would be associated with reduced mortality risk. In addition, we investigated the joint effects of weight training and aerobic exercise on mortality outcomes.

## Methods

This study used data from the National Institutes of Health-American Association of Retired Persons (NIH-AARP) Diet and Health Study, a cohort of 566 398 AARP members who completed a mailed entry questionnaire regarding demographics, medical history and dietary behaviours in 1995–96.^19^ Further information on the cohort is available elsewhere.^19^ Participants who completed the entry questionnaire were invited to complete a questionnaire at two subsequent time points: 1996–97 (not used in this analysis) and 2004–05.

The 2004–05 questionnaire collected detailed information on lifestyle and physical activity (including weight training) and was completed by 313 791 participants, who ranged in age from 59 to 82 years at the time of completion. These participants form the basis of the current analysis. The following exclusion criteria were then applied (in order): proxy respondents (*n *= 20 054); participants who reported being unable to walk (*n* = 7418); participants who did not complete the weight training question (*n* = 13 297); and participants with missing data for any confounders included in the analyses (*n* = 56 683). After these exclusions, 216 339 participants remained and were included in the current analyses (Supplementary Figure S1, available as Supplementary data at *IJE* online).

### Measurement of weight training and aerobic exercise

In the 2004–05 questionnaire, participants were asked to report the amount of time per week spent performing different physical activities and exercises, including weight training, over the past 12 months. The full definition of weight training provided to participants in the questionnaire was ‘weight training or lifting (include free weights and machines)’. Participants could choose from 10 different categories: none, 5 min, 15 min, 30 min, 1 h, 1.5 h, 2–3 h, 4–6 h, 7–10 h and >10 h. Similar questionnaires demonstrate acceptable validity and reliability when measuring weight training and muscle-strengthening exercises (intraclass correlation coefficients ∼0.5–0.6).^20^^,^^21^ To avoid sparsely populated categories, these data were categorized as: none, 5–15 min, 30 min, 1 h, 1.5 h, 2–3 h and 4 h and greater in the primary analyses examining the associations between weight training and mortality risk; and none, 5–30 min, 1–1.5 h and >2 h in the joint effects analyses.

Metabolic equivalents of task-h (MET-h) per week of leisure-time aerobic exercise were calculated based on time spent per week in each of: jogging, tennis, golf, swimming, cycling, walking for exercise and other aerobic activity.^19^ To facilitate calculation of MET-h/week of aerobic exercise, the 10 categories described above were converted to a continuous ‘h/week’ measure, with the mid-point used when the response option was a range, and a MET-value was assigned to each activity. Total MET-h/week of aerobic exercise was categorized as none (0 MET-h/week), with the remaining participants classified into three categories of roughly equal size which were defined as low (0.1 to <10.75), medium (10.75 to <29) and high (>29).

### Ascertainment of outcome

Mortality data from the Social Security Administration Death Master File and the National Data Index Plus were used to ascertain mortality status. We used Surveillance, Epidemiology, End Result (SEER) codes for underlying and contributing causes of death to identity CVD deaths (codes 50060 to 50110) and cancer deaths (codes 20010 to 37000). Follow-up time began at the 2004–05 questionnaire completion date and ended at death or the time of the last available mortality extract (31 December 2019), whichever occurred first.

### Statistical analysis

Participant characteristics were categorized using descriptive statistics. Cox proportional hazards regression was used to estimate hazard ratios (HRs) and associated 95% confidence intervals (CIs) for the associations between weight training and all-cause, CVD and cancer mortality, after adjusting for confounders. We used a directed acyclic graph (DAG) to identify confounders based on the determinants of weight training and mortality risk factors. The following variables were included as confounders in all models: age, sex, education, race, body mass index (BMI), alcohol consumption, self-reported health status (as a proxy for comorbidity and overall health) and healthy eating index,^22^ all of which were based on self-reported information from the 1994–95 entry questionnaire; and leisure-time aerobic exercise and cigarette smoking, which were taken from the 2004–05 questionnaire (see Table 1 for further information about these variables). Interaction terms were added to the models and global Wald tests were conducted to determine whether the associations between weight training and mortality risk differed by sex or by concurrent leisure-time aerobic exercise.

**Table 1.** Characteristics of the participants in the NIH-AARP Diet and Health study who returned the follow-up questionnaire, by levels of weight training (*n *=* *216 339)

	Weight training/week						
Characteristic	None	5–15 min	30 min	1 h	1.5 h	2–3 h	4+ h
*n* (%)	162 246 (75.0)	9800 (4.5)	11 223 (5.2)	11 687 (5.4)	8188 (3.8)	8792 (4.1)	4403 (2.0)
Age (years), 2004–05, mean (SD)	70.1 (5.4)	69.4 (5.5)	69.5 (5.4)	69.4 (5.4)	69.1 (5.2)	68.9 (5.3)	68.8 (5.2)
Sex (%)							
Men	57.0	62.2	59.9	61.4	62.3	64.3	69.7
Women	43.0	37.8	40.1	38.6	37.7	35.7	30.3
Race (%)							
White	94.0	93.5	94.3	94.7	95.2	94.9	92.6
Black	3.1	3.1	2.7	2.3	1.9	1.9	3.7
Hispanic	1.5	1.4	1.5	1.5	1.5	1.7	2.4
Other	1.4	2.0	1.6	1.4	1.3	1.5	1.3
Smoking status, 2004–05 (%)							
Never smoker	40.3	41.9	41.2	39.9	38.5	38.6	38.1
Former smoker, quit 10+ years ago	42.5	46.3	47.0	48.1	49.3	49.5	49.1
Former smoker, quit 5–9 year ago	3.4	2.9	2.7	3.0	3.2	2.8	2.5
Former smoker, quit <5 years ago	2.8	2.1	2.0	2.0	2.2	1.9	2.3
Former smoker, time since quitting unknown	4.3	3.7	4.1	4.1	4.0	4.3	4.8
Current smoker	6.7	3.1	3.1	2.8	2.6	2.9	3.2
Education level, 1995–96 (%)							
<12 years	4.0	1.8	1.6	1.5	1.7	1.4	2.7
12 years or completed high school	19.5	10.9	11.2	10.0	9.7	9.6	12.8
Post high-school training	10.4	7.7	7.4	7.9	6.6	7.1	7.5
Some college	24.2	20.7	22.0	21.8	21.8	21.1	23.4
College graduate	41.9	58.9	57.8	58.8	60.2	60.8	53.6
Self-reported health, 1995–96 (%)							
Excellent	16.9	22.0	24.6	27.1	30.1	32.5	33.7
Very good	37.9	40.0	41.7	41.3	41.6	41.1	39.4
Good	35.8	31.0	28.1	27.0	24.3	22.6	22.4
Fair	8.6	6.4	5.3	4.2	3.6	3.6	4.2
Poor	0.8	0.6	0.4	0.4	0.3	0.3	0.3
Body mass index (kg/m^2^), 1995–96 (%)							
<18.5	0.9	1.1	1.0	1.0	1.2	1.1	1.0
18.5–<25	34.0	42.0	42.9	44.8	45.4	45.8	43.0
25–<30	43.0	40.9	41.8	40.6	40.2	40.3	42.8
30–<35	16.0	12.1	11.1	10.8	10.5	10.0	10.2
35+	6.1	4.0	3.3	2.8	2.6	2.8	2.9
Body mass index (kg/m^2^), 1995–96, mean (SD)	27.1 (4.9)	26.2 (4.6)	26.0 (4.6)	25.9 (4.2)	25.8 (4.1)	25.8 (4.1)	25.9 (4.8)
Alcohol intake (g/day), 1995–96, mean (SD)	12.8 (34.7)	12.6 (29.6)	12.1 (28.0)	12.3 (27.8)	12.9 (27.0)	12.9 (28.8)	14.6 (35.3)
Healthy Eating Index score, 1995–96, mean (SD)	67.5 (9.5)	69.7 (8.9)	70.4 (8.8)	70.8 (8.6)	71.3 (8.5)	71.1 (8.6)	70.4 (8.8)
Aerobic leisure-time exercise (MET-h/week), 2004–05, mean (SD)	20.2 (26.6)	25.0 (25.0)	31.8 (27.2)	38.2 (31.0)	40.6 (31.7)	49.7 (38.3)	97.0 (84.5)
Aerobic leisure-time exercise (MET-h/week), 2004–05 (%)							
0	13.6	1.7	1.5	1.3	1.3	1.4	1.9
0.1–<10.75	31.9	31.0	16.6	9.9	6.5	5.1	3.9
10.75–<29	30.3	36.3	39.2	36.5	35.0	24.9	11.8
29+	24.2	30.9	42.6	52.4	57.2	68.6	82.4

MET, metabolic equivalent of task; NIH-AARP, National Institutes of Health-American Association of Retired Persons; SD, standard deviation.

Two sensitivity analyses were performed to evaluate risk of bias associated with reverse causation and potential confounding by chronic conditions: (i) excluding participants who self-reported being diagnosed with cancer, angina, heart attack, coronary disease or stroke prior to follow-up questionnaire completion; and (ii) censoring deaths within the first 2 years of follow-up. Further sensitivity analyses were conducted to assess the impact of selection bias induced by: (i) a large proportion of the 1994–95 entry cohort not completing the 2004–05 questionnaire; and (ii) participants being excluded from the analytical sample due to missing data. We first compared the characteristics of the analytical sample with participants who did not complete the follow-up questionnaire and participants who were excluded due to missing data. Second, we predicted the likelihood of being in the analytical sample using the variables reported in Supplementary Table S1 (available as Supplementary data at *IJE* online), then used these values as weights in an inverse probability weighting-based analysis to account for the potential impact of exclusions.

We investigated the joint associations of weight training and aerobic exercise by creating joint categories of weight training and aerobic exercise consisting of all combinations of four categories of weight training and four categories of leisure-time aerobic exercise.

Proportional hazards assumptions were tested using the Schoenfeld residuals technique; no meaningful violations were observed. All statistical analyses were conducted using Stata v15.1 (StataCorp, College Station, TX). Reporting follows the STROBE guidelines.^23^

## Results

The mean age of participants was 69.9 years (standard deviation = 5.4 years) at the time of the 2004–05 follow-up questionnaire completion. There was a higher proportion of men (58%) and White participants (94%), and 46% were college graduates. There were 79 107 (37%) deaths during the follow-up period (median follow-up time = 15 years), of which 56% and 31% were due to CVD and cancer, respectively.

Approximately 25% of participants reported doing weight training (Table 1). Weight training was more prevalent among younger participants, men, participants with a BMI in the 18.5–<25 kg/m^2^ range, participants with higher levels of aerobic exercise, participants with higher education levels and participants who self-reported their health as excellent.

### Weight training and mortality risk

Compared with participants who did not perform any weight training, participants who performed any weight training had a 6% lower risk of all-cause mortality (HR = 0.94; 95% CI = 0.93–0.96), a 8% lower risk of CVD mortality (HR = 0.92; 95% CI = 0.90–0.95) and a 5% lower risk of cancer mortality (HR = 0.95; 95% CI = 0.92–0.98) (Table 2). More time spent in weight training was associated with only marginally greater risk reductions.

**Table 2.** Associations between weight training and all-cause, cardiovascular disease and cancer mortality overall and among men and women (*n *=* *216 339)

		All-cause mortality		CVD mortality			Cancer mortality	
Weight training	Person-years	Deaths	HR (95% CI), *P*^a^	Deaths	HR (95% CI), *P*^a^		Deaths	HR (95% CI), *P*^a^
**All participants**								
No weight training	2 046 616	62 550	1.00 (Reference)	35 217	1.00 (Reference)		19 314	1.00 (Reference)
Some weight training	715 998	16 557	0.94 (0.93–0.96), <0.001	8953	0.92 (0.90–0.95), <0.001		5459	0.95 (0.92–0.98), 0.001
Categories (none is reference)								
5–15 min/week	127 589	3199	0.95 (0.91–0.98), 0.003	1746	0.92 (0.88–0.97), 0.001		997	0.93 (0.87–0.99), 0.030
30 min/week	147 705	3564	0.95 (0.92–0.98), 0.003	1954	0.94 (0.89–0.98), 0.005		1173	0.97 (0.91–1.03), 0.292
1 h/week	154 615	3653	0.97 (0.93–1.00), 0.045	1964	0.94 (0.90–0.98), 0.008		1178	0.95 (0.89–1.01), 0.081
1.5 h/week	109 111	2421	0.94 (0.90–0.98), 0.002	1309	0.93 (0.87–0.98), 0.007		801	0.93 (0.86–0.99), 0.036
2–3 h/week	118 202	2474	0.91 (0.88–0.95), <0.001	1297	0.88 (0.83–0.93), <0.001		883	0.96 (0.90–1.03), 0.248
4+ h/week	58 776	1246	0.92 (0.87–0.97), 0.004	683	0.92 (0.85–0.99), 0.027		427	0.91 (0.83–1.01), 0.066
**Men**								
No weight training	1 136 981	39 247	1.00 (Reference)	22 586	1.00 (Reference)		12 405	1.00 (Reference)
Some weight training	437 390	11 851	0.97 (0.95–0.99), 0.012	6599	0.96 (0.93–0.99), 0.005		3874	0.96 (0.92–1.00), 0.032
Categories (none is reference)								
5–15 min/week	77 216	2277	0.97 (0.93–1.01), 0.137	1269	0.94 (0.89–1.00), 0.041		684	0.92 (0.85–0.99), 0.025
30 min/week	86 204	2460	0.98 (0.94–1.02), 0.344	1.372	0.96 (0.91–1.02), 0.161		829	1.01 (0.94–1.09), 0.692
1 h/week	92 664	2598	1.01 (0.97–1.05), 0.781	1442	0.99 (0.94–1.04), 0.696		844	0.99 (0.92–1.06), 0.696
1.5 h/week	66 505	1729	0.96 (0.91–1.01), 0.102	979	0.97 (0.91–1.04), 0.373		559	0.92 (0.85–1.01), 0.067
2–3 h/week	74 479	1821	0.95 (0.90–0.99), 0.030	991	0.93 (0.87–0.99), 0.024		632	0.96 (0.89–1.05), 0.380
4+ h/week	40 321	969	0.94 (0.88–1.01), 0.075	546	0.96 (0.88–1.04), 0.334		326	0.91 (0.82–1.02), 0.112
**Women**								
No weight training	906 635	23 303	1.00 (Reference)	12 631	1.00 (Reference)		6909	1.00 (Reference)
Some weight training	278 608	4703	0.88 (0.85–0.91), <0.001	2354	0.84 (0.80–0.88), <0.001		1585	0.91 (0.86–0.97), 0.002
Categories (none is reference)								
5–15 min/week	50 374	922	0.90 (0.84–0.96), 0.002	477	0.88 (0.80–0.96), 0.006		313	0.97 (0.86–1.08), 0.542
30 min/week	61 500	1104	0.89 (0.83–0.94), <0.001	582	0.88 (0.81–0.96), 0.003		344	0.87 (0.78–0.97), 0.013
1 h/week	61 950	1055	0.88 (0.82–0.93), <0.001	522	0.83 (0.75–0.90), <0.001		334	0.86 (0.77–0.96), 0.009
1.5 h/week	42 605	692	0.89 (0.82–0.96), 0.002	330	0.81 (0.73–0.91), <0.001		242	0.93 (0.82–1.06), 0.287
2–3 h/week	43 723	653	0.84 (0.77–0.90), <0.001	306	0.76 (0.67–0.85), <0.001		251	0.95 (0.84–1.09), 0.484
4+ h/week	18 455	277	0.86 (0.76–0.97), 0.012	137	0.81 (0.68–0.96), 0.013		101	0.93 (0.76–1.13), 0.446

CI, confidence interval; CVD, cardiovascular disease; HR, hazard ratio; kg, kilogram; m, metre.

a Adjusted for the following confounders: age, sex, education, race, body mass index, leisure-time aerobic exercise, cigarette smoking, alcohol consumption, Healthy Eating Index, self-reported health status.

The associations between weight training and the risks of all-cause and CVD mortality, but not cancer mortality, were stronger in women compared with men (*P_interaction_* <0.001 for all-cause and CVD mortality in all analyses, *P_interaction_* for cancer mortality = 0.339 and 0.141 in None/Some and categorical weight training analyses, respectively; Table 2). Among women, performing any weight training was associated with 12%, 16% and 9% decreased risks of all-cause, CVD and cancer mortality, respectively, compared with doing none. The corresponding risk reductions among men were 3%, 4% and 4%.

The results of sensitivity analyses investigating the potential impact of reverse causation, confounding by chronic conditions and selection bias, were not meaningfully different from the original analyses (Supplementary Table S2, available as Supplementary data at *IJE* online).

### Joint effects of weight training and aerobic exercise on mortality risk

The association between weight training and mortality risk appeared to vary by level of aerobic leisure-time exercise, although the interaction term for weight training and aerobic exercise was statistically significant for CVD mortality (*P_Interaction_* *= 0.014*) but not all-cause mortality (*P_Interaction_* = 0.264) or cancer mortality (*P_interaction_* = 0.586).

**Figure 1.**
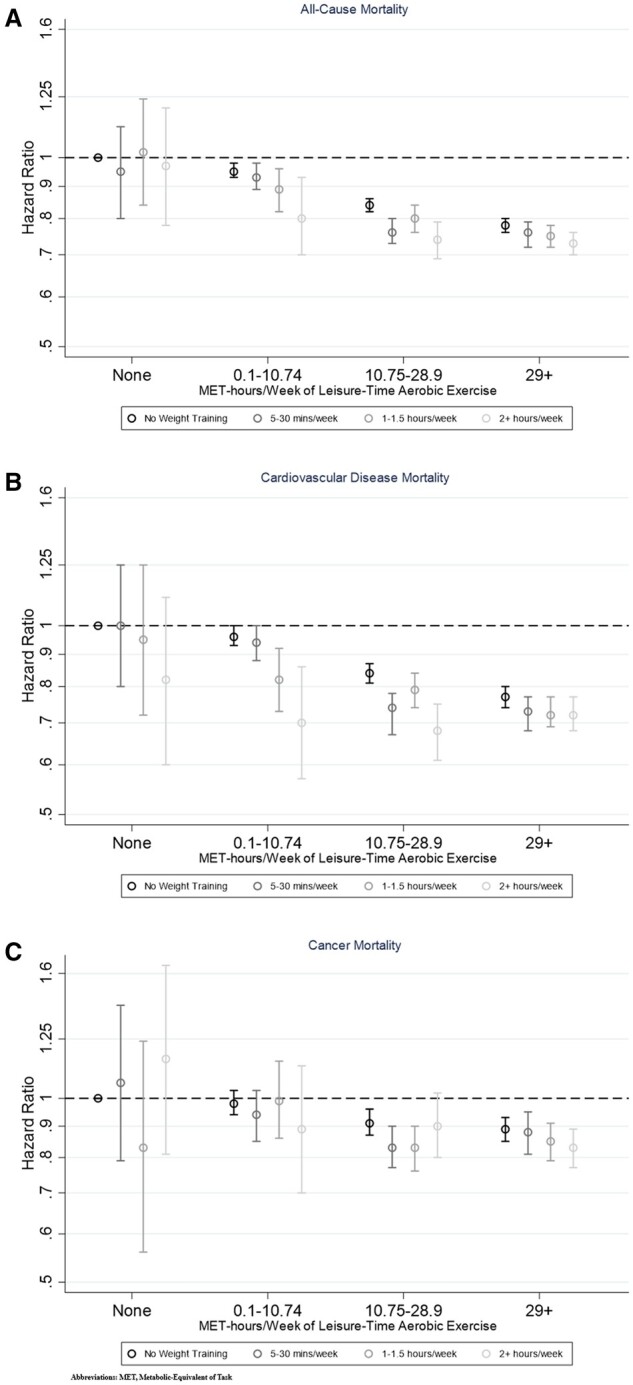
Associations between joint categories of weight training and moderate to leisure-time aerobic exercise and the risks of (A) all-cause mortality, (B) cardiovascular disease mortality and (C) cancer mortality. MET-h, metabolic equivalent of task-h

There was no evidence that weight training was associated with all-cause or cancer mortality among participants who did not report performing any aerobic leisure-time exercise, and only weak evidence of a potential reduction in CVD mortality was observed (Figure 1; Supplementary Table S3, available as Supplementary data at *IJE* online). Similarly, engaging in weight training appeared to confer little mortality benefit among participants who reported the highest levels of aerobic exercise. For example, HRs for all-cause mortality among participants in the highest category aerobic exercise were 0.78 (95% CI = 0.76–0.80) for no weight training and 0.73 (95% CI = 0.70–0.76) for 2+ h/week of weight training, compared with participants who performed neither weight training nor aerobic exercise.

Increasing min/week of weight training did appear to provide additional mortality benefits to those provided by aerobic exercise among participants who performed low-to-intermediate levels of aerobic activity. For all-cause mortality, among those who performed 0.1–<10.75 MET-h/week of aerobic exercise, the HRs decreased from 0.95 (95% CI = 0.93–0.98) for no weight training to 0.80 (95% CI = 0.70–0.93) for the highest level of weight training, and the corresponding HRs among participants who performed 10.75–<29 75 MET-h/week were 0.84 (95% CI = 0.82–0.86) and 0.74 (95% CI = 0.69–0.79). Larger risk reductions were observed for CVD mortality than for cancer mortality.

Increasing levels of aerobic exercise were associated with increasingly greater risk reductions for all three mortality outcomes, across all levels of weight training.

## Discussion

In this large cohort study of older adults, engaging in any amount of weight training was associated with a 6% lower risk of all-cause mortality, 8% lower risk of CVD mortality and 5% lower risk of cancer mortality, compared with those who did none. Larger risk reductions were observed for women than men, particularly for all-cause mortality (3% in men vs 12% in women) and CVD mortality (4% in men vs 16% in women). Engaging in high levels of both weight training and aerobic leisure-time exercise was associated with greater mortality benefits compared with performing either of these activities alone, particularly among participants who performed low-to-intermediate levels of aerobic exercise.

Our results are consistent with previous studies on the association between muscle-strengthening exercises and mortality risk and provide strong evidence for this association among older adults. Consistent with our study, recent research indicates engaging in muscle-strengthening exercises reduces the risk of all-cause mortality by 10–15% compared with doing none, and also reduces the risk of both CVD and cancer mortality.^14^^,^^16^^,^^17^^,^^24^ Given fewer than 20% of adults in the USA, Europe and Australia meet current muscle-strengthening activity guidelines,^25–27^ and that this figure is even lower among older adults,^4^ our study supports a greater emphasis on increasing the prevalence of muscle-strengthening exercises among older adults.

Our study provides additional insights into whether the association between muscle-strengthening exercises and mortality differs by sex, with stronger associations for all-cause mortality and CVD mortality observed among women than men. Whileeas one previous study also found weight training was associated with larger mortality risk reductions among women than men,^17^ most studies have reported no effect modification by sex^18^^,^^28–30^ and a potential biologically plausible mechanism is not clear. Future research should further investigate whether the association between muscle-strengthening exercises and mortality differs by sex and potential biological mechanisms. Regardless, our study provides robust evidence that weight training is associated with lower mortality risk among older women, a group among whom the prevalence of participation in muscle-strengthening exercises is particularly low.^4^

The large number of participant deaths in this cohort allowed us to investigate the joint effects of weight training and aerobic exercise with greater depth and precision than previous studies. Consistent with previous research, engaging in both exercise types provided the greatest mortality benefits,^30–33^ supporting the recommendations of physical activity guidelines to perform muscle-strengthening exercises in addition to aerobic MVPA.^1^ Weight training was associated with mortality benefits additional to those conferred by aerobic exercise among older adults who engaged in low-to-intermediate levels of aerobic activity. However, it was not associated with all-cause or cancer mortality among participants who performed no aerobic exercise, and only weakly associated with CVD mortality. Weight training was not associated with additional mortality risk reductions among participants with the highest levels of aerobic exercise, perhaps due to this group already being at lower mortality risk. One possible explanation for these findings is that muscle-strengthening exercises, when performed in combination with aerobic exercise, may enhance the health benefits conveyed by aerobic exercise. This is supported by evidence from clinical trials which indicates that, for a range of anthropometric and cardiometabolic markers, the benefits gained by performing a combination of muscle-strengthening exercise and aerobic exercise are greater than those seen for aerobic exercise alone, even when the benefits of muscle-strengthening exercises alone may be modest.^10^^,^^11^^,^^34^ Similar findings have been observed for mortality due to a range of causes.^35^ There are also likely to be cross-over effects between aerobic and muscle-strengthening exercises, e.g. weight-bearing aerobic exercises (e.g. running) may improve muscle and bone strength, and some muscle-strengthening exercises may improve cardiovascular fitness.

Our study is one of the few large cohorts to have information on min/week of muscle-strengthening exercise, so provides novel and important insights into the dose-response relationship. We found increasing levels of weight training were associated with only marginally greater risk reductions. A recent dose-response meta-analysis of four studies found a ‘U-shaped’ relationship between resistance training volume and all-cause mortality risk, with the largest reduction (33%) observed at 60 min/week and greater volumes associated with smaller or no risk reductions.^16^ Although somewhat in contrast, both studies suggest large volumes of muscle-strengthening exercises are not needed to receive the optimal mortality benefits.

Strengths of this study include its prospective design, large sample size and long follow-up period, recording a large number of deaths. Having information about min/week spent performing weight training meant we could look at the dose-response relationship with mortality in more detail than most previous studies, which have only measured frequency of muscle-strengthening exercises (e.g. number of sessions/week).

Limitations included the use of self-reported weight training data, which are susceptible to measurement error. Few studies have investigated the validity and reliability of self-reported weight training,^36^ and participants may have reported time spent at the gym rather than time engaged in weight training activity specifically. We lacked data on the weight, number of repetitions and intensity of weight training, as well as information about other forms of muscle-strengthening exercises (including those performed in other domains, such as occupational settings). Lacking information about the frequency of weight training limited direct evaluation of the current physical activity guidelines, which recommend two or more sessions/week rather than a specific number of minutes. We also only had information about weight training at a single point in time. Future cohort studies with more detailed measures of muscle-strengthening exercises, assessed longitudinally and with valid and reliable tools, would provide stronger evidence about the association with mortality risk. Finally, participants were predominantly older, White adults who were more likely to have higher education levels and better health than the general population, and it is not known if the observed results are generalizable to populations with different sociodemographic characteristics.

Our results indicate engaging in muscle-strengthening exercises such as weight training has mortality benefits for older adults, even at low levels. Given the low prevalence in the general population, and older adults in particular,^4^^,^^25^ further emphasis should be placed on the benefits of muscle-strengthening activities for mortality risk reduction in public health programmes.

## Ethics approval

The NIH-AARP Diet and Health Study was approved by the Special Studies Institutional Review Board of the National Cancer Institute. All participants gave informed consent.

## Supplementary Material

dyae074_Supplementary_Data

## Data Availability

Data for the NIH-AARP Diet and Health Study are maintained by the National Cancer Institute, Division of Cancer Epidemiology and Genetics. Data described in the manuscript, a code book and analytical code will be made available upon request pending study approval from the NIH-AARP Diet and Health Study Steering Committee. Further details are provided at [https://www.nihaarpstars.com/].
