# Supplementary material for: Weight training and risk of all-cause, cardiovascular disease and cancer mortality among older adults
Source: Int J Epidemiol. 2024 Jun 3;53(3):dyae074. doi: 10.1093/ije/dyae074 (PMC11147802; doi:10.1093/ije/dyae074)

**SUPPLEMENTARY MATERIAL**

**Weight training and risk of all-cause, cardiovascular disease and cancer mortality among older adults**

**Supplementary Table S1: Baseline characteristics of included and excluded participants**

|  | **Did not Complete Follow-Up Questionnaire** | **Completed Follow-Up Questionnaire but Excluded due to missing data** | **Included in Analysis** |
| --- | --- | --- | --- |
| **n** | **252,607** | **97,452** | **216,339** |
| **Age (years), mean (SD)** | 61.9 (5.4) | 62.0 (5.3) | 61.2 (5.4) |
| **Sex** |  |  |  |
| Men | 61.6 | 59.1 | 58.4 |
| Women | 38.4 | 40.9 | 41.6 |
| **Race (%)** |  |  |  |
| White | 91.3 | 92.2 | 94.1 |
| Black | 4.7 | 4.1 | 3.0 |
| Hispanic | 2.2 | 2.0 | 1.5 |
| Other | 1.8 | 1.7 | 1.4 |
| **Education Level (%)** |  |  |  |
| <12 years | 9.2 | 6.9 | 3.5 |
| 12 years or completed high school | 23.3 | 19.4 | 17.3 |
| Post high-school training | 10.7 | 9.8 | 9.6 |
| Some college | 24.4 | 22.4 | 23.5 |
| College graduate | 32.4 | 41.5 | 46.1 |
| **Self-Reported Health (%)** |  |  |  |
| Excellent | 13.6 | 17.6 | 19.5 |
| Very Good | 31.2 | 35.2 | 38.7 |
| Good | 36.5 | 34.9 | 33.4 |
| Fair | 15.3 | 10.8 | 7.6 |
| Poor | 3.4 | 1.5 | 0.7 |
| **Body Mass Index (kg/m^2^), mean (SD)** | 27.3 (5.3) | 27.2 (5.2) | 26.8 (4.8) |
| **Alcohol Intake (grams per day), mean (SD)** | 13.9 (42.8) | 12.6 (36.3) | 12.8 (33.3) |
| **Healthy Eating Index score, mean (SD)** | 66.6 (9.8) | 68.1 (9.5) | 68.3 (9.4) |
| **Participation in Physical Activity for >=20 mins in last 12 months, baseline** |  |  |  |
| Never | 6.6 | 4.3 | 2.9 |
| Rarely | 15.8 | 12.6 | 12.4 |
| 1-3 times/month | 13.7 | 12.8 | 14.0 |
| 1-2 times/week | 20.8 | 21.1 | 22.6 |
| 3-4 times/week | 24.9 | 27.9 | 28.4 |
| 5+ times/week | 18.1 | 21.4 | 19.8 |

Abbreviations: kg, Kilogram; m, Metre; SD, Standard Deviation

**Supplementary Table S2. Results of sensitivity analyses investigating the associations between weight training and all-cause, cardiovascular disease and cancer mortality**

|  | **Original analysis (n=216,339 in analysis)** | **Excluding those with <24 months of follow-up (n=210,973 in analysis)** | **Excluding those who reported a prior cancer or CVD diagnosis (n=126,801 in analysis)** | **Inverse Probability Weighting** |
| --- | --- | --- | --- | --- |
|  | **HR (95% CI), *p-value*^a^** | **HR (95% CI), *p-value*^a^** | **HR (95% CI), *p-value*^a^** | **HR (95% CI), *p-value*^a^** |
| **All-Cause Mortality** |  |  |  |  |
| No weight training | *1.00 (Reference)* | *1.00 (Reference)* | *1.00 (Reference)* | *1.00 (Reference)* |
| Some weight training | 0.94 (0.93, 0.96), *<0.001* | 0.95 (0.93, 0.96), *<0.001* | 0.93 (0.91, 0.96), *<0.001* | 0.95 (0.93, 0.97), *<0.001* |
| *Categories (None is Reference)* |  |  |  |  |
| 5-15 minutes per week | 0.95 (0.91, 0.98), *0.003* | 0.94 (0.89, 0.99), *0.007* | 0.95 (0.92, 0.99), *0.018* | 0.95 (0.92, 0.99), *0.009* |
| 30 minutes per week | 0.95 (0.92, 0.98), *0.003* | 0.94 (0.89, 0.98), *0.007* | 0.95 (0.92, 0.99), *0.008* | 0.95 (0.92, 0.98), *0.004* |
| 1 hour per week | 0.97 (0.93, 1.00), *0.045* | 0.97 (0.92, 1.01), *0.027* | 0.96 (0.93, 1.00), *0.159* | 0.98 (0.94, 1.01), *0.169* |
| 1.5 hours per week | 0.94 (0.90, 0.98), *0.002* | 0.93 (0.88, 0.99), *0.005* | 0.94 (0.90, 0.98), *0.019* | 0.95 (0.91, 0.99), *0.019* |
| 2–3 hours per week | 0.91 (0.88, 0.95), <*0.001* | 0.90 (0.85, 0.96), <*0.001* | 0.92 (0.88, 0.96), *0.001* | 0.92 (0.88, 0.96), <*0.001* |
| 4+ hours per week | 0.92 (0.87, 0.97), *0.004* | 0.89 (0.82, 0.97), *0.006* | 0.92 (0.87, 0.98), *0.005* | 0.91 (0.86, 0.97), *0.003* |
|  |  |  |  |  |
| **CVD Mortality** |  |  |  |  |
| No weight training | *1.00 (Reference)* | *1.00 (Reference)* | *1.00 (Reference)* | *1.00 (Reference)* |
| Some weight training | 0.92 (0.90, 0.95), *<0.001* | 0.92 (0.90, 0.95), *<0.001* | 0.89 (0.86, 0.93), <*0.001* | 0.92 (0.90, 0.95), *<0.001* |
| *Categories (None is Reference)* |  |  |  |  |
| 5-15 minutes per week | 0.92 (0.88, 0.97), *0.001* | 0.93 (0.89, 0.98), *0.005* | 0.88 (0.82, 0.95), *0.001* | 0.93 (0.88, 0.98), *0.004* |
| 30 minutes per week | 0.94 (0.89, 0.98), *0.005* | 0.94 (0.89, 0.98), *0.007* | 0.90 (0.84, 0.96), *0.002* | 0.93 (0.89, 0.98), *0.007* |
| 1 hour per week | 0.94 (0.90, 0.98), *0.008* | 0.93 (0.89, 0.98), *0.003* | 0.92 (0.86, 0.98), *0.014* | 0.95 (0.90, 0.99), *0.025* |
| 1.5 hours per week | 0.93 (0.87, 0.98), *0.007* | 0.93 (0.88, 0.98), *0.010* | 0.92 (0.85, 1.00), *0.046* | 0.93 (0.88, 0.99), *0.017* |
| 2–3 hours per week | 0.88 (0.83, 0.93), <*0.001* | 0.88 (0.83, 0.94), *<0.001* | 0.89 (0.82, 0.96), *0.003* | 0.87 (0.82, 0.92), <*0.001* |
| 4+ hours per week | 0.92 (0.85, 0.99), *0.027* | 0.91 (0.85, 0.99), *0.027* | 0.82 (0.73, 0.92), *0.001* | 0.92 (0.84, 0.99), *0.034* |
|  |  |  |  |  |
| **Cancer Mortality** |  |  |  |  |
| No weight training | *1.00 (Reference)* | *1.00 (Reference)* | *1.00 (Reference)* | *1.00 (Reference)* |
| Some weight training | 0.95 (0.92, 0.98), *0.001* | 0.96 (0.93, 0.99), *0.010* | 0.95 (0.90, 0.99), *0.027* | 0.95 (0.92, 0.98), *0.002* |
| *Categories (None is Reference)* |  |  |  |  |
| 5-15 minutes per week | 0.93 (0.87, 0.99), *0.030* | 0.94 (0.88, 1.01), *0.086* | 0.95 (0.87, 1.05), *0.343* | 0.93 (0.87, 0.99), *0.028* |
| 30 minutes per week | 0.97 (0.91, 1.03), *0.292* | 0.98 (0.93, 1.05), *0.630* | 0.93 (0.85, 1.01), *0.099* | 0.97 (0.91, 1.03), *0.368* |
| 1 hour per week | 0.95 (0.89, 1.01), *0.081* | 0.96 (0.90, 1.02), *0.153* | 0.99 (0.90, 1.08), *0.761* | 0.96 (0.90, 1.02), *0.167* |
| 1.5 hours per week | 0.93 (0.86, 0.99), *0.036* | 0.93 (0.87, 1.01), *0.076* | 0.90 (0.81, 1.00), *0.060* | 0.94 (0.87, 1.01), *0.114* |
| 2–3 hours per week | 0.96 (0.90, 1.03), *0.248* | 0.98 (0.91, 1.05), *0.546* | 0.99 (0.90, 1.10), *0.875* | 0.97 (0.90, 1.04), *0.353* |
| 4+ hours per week | 0.91 (0.83, 1.01), *0.066* | 0.92 (0.83, 1.02), *0.118* | 0.89 (0.77, 1.02), *0.092* | 0.89 (0.81, 0.99), *0.026* |

Abbreviations: CI, Confidence Interval; CVD, Cardiovascular disease; HR, Hazard Ratio

^a^ Adjusted for the following confounders: age, sex, education, race, body mass index, leisure-time aerobic activity, cigarette smoking, alcohol consumption, health eating index, self-reported health status

**Supplementary Table S3. Associations between joint categories of weight training and leisure-time aerobic exercise and the risks of all-cause, cardiovascular disease and cancer mortality (n= 216,339)**

|  | **Aerobic Exercise (MET-Hours per Week)** | | | |
| --- | --- | --- | --- | --- |
|  | **None** | **0.1 to <10.75** | **10.75 to <29** | **29+** |
| **Weight training** | **HR (95% CI), *p-value*^a^** | **HR (95% CI), *p-value*^a^** | **HR (95% CI), *p-value*^a^** | **HR (95% CI), *p-value*^a^** |
| **ALL-CAUSE MORTALITY** |  |  |  |  |
| **None** | *1.00 (Reference)* | 0.95 (0.93, 0.98)*, <0.001* | 0.84 (0.82, 0.86)*, <0.001* | 0.78 (0.76, 0.80)*, <0.001* |
| **5-30 minutes per week** | 0.95 (0.80, 1.12)*, 0.538* | 0.93 (0.89, 0.98)*, 0.006* | 0.76 (0.73, 0.80)*, <0.001* | 0.76 (0.72, 0.79)*, <0.001* |
| **1-1.5 hours per week** | 1.02 (0.84, 1.24)*, 0.838* | 0.89 (0.82, 0.96)*, 0.005* | 0.80 (0.76, 0.84)*, <0.001* | 0.75 (0.72, 0.78)*, <0.001* |
| **2+ hours per week** | 0.97 (0.78, 1.20)*, 0.751* | 0.80 (0.70, 0.93)*, 0.002* | 0.74 (0.69, 0.79)*, <0.001* | 0.73 (0.70, 0.76)*, <0.001* |
|  |  |  |  |  |
| **CVD MORTALITY** |  |  |  |  |
| **None** | *1.00 (Reference)* | 0.96 (0.93, 1.00)*, 0.027* | 0.84 (0.81, 0.87)*, <0.001* | 0.77 (0.74, 0.80)*, <0.001* |
| **5-30 minutes per week** | 1.00 (0.80, 1.25)*, 0.999* | 0.94 (0.88, 1.00)*, 0.066* | 0.74 (0.69, 0.78)*, <0.001* | 0.73 (0.68, 0.77)*, <0.001* |
| **1-1.5 hours per week** | 0.95 (0.72, 1.25)*, 0.713* | 0.82 (0.73, 0.92)*, 0.001* | 0.79 (0.74, 0.84)*, <0.001* | 0.72 (0.69, 0.77)*, <0.001* |
| **2+ hours per week** | 0.82 (0.60, 1.11)*, 0.200* | 0.70 (0.57, 0.86)*, 0.001* | 0.68 (0.61, 0.75)*, <0.001* | 0.72 (0.68, 0.77)*, <0.001* |
|  |  |  |  |  |
| **CANCER MORTALITY** |  |  |  |  |
| **None** | *1.00 (Reference)* | 0.98 (0.94, 1.03)*, 0.480* | 0.91 (0.87, 0.96)*, <0.001* | 0.89 (0.85, 0.93)*, <0.001* |
| **5-30 minutes per week** | 1.06 (0.79, 1.42)*, 0.715* | 0.94 (0.85, 1.03)*, 0.167* | 0.83 (0.77, 0.90)*, <0.001* | 0.88 (0.81, 0.95)*, 0.001* |
| **1-1.5 hours per week** | 0.83 (0.56, 1.24)*, 0.366* | 0.99 (0.86, 1.15)*, 0.910* | 0.83 (0.76, 0.90)*, <0.001* | 0.85 (0.79, 0.91)*, <0.001* |
| **2+ hours per week** | 1.16 (0.81, 1.65)*, 0.417* | 0.89 (0.70, 1.13)*, 0.337* | 0.90 (0.80, 1.02)*, 0.103* | 0.83 (0.77, 0.89)*, <0.001* |

Abbreviations: CI, Confidence Interval; CVD: Cardiovascular disease; HR: Hazard Ratio; MET, Metabolic-Equivalent of Task

^a^Adjusted for the following confounders: age, sex, education, race, body mass index, cigarette smoking, alcohol consumption, healthy eating index, self-reported health status

**Supplementary Figure S1: Flow chart of National Institutes of Health-American Association of Retired Persons (NIH-AARP) Cohort Study participants included in an analysis investigating the associations between weight training and all-cause, cardiovascular disease mortality**


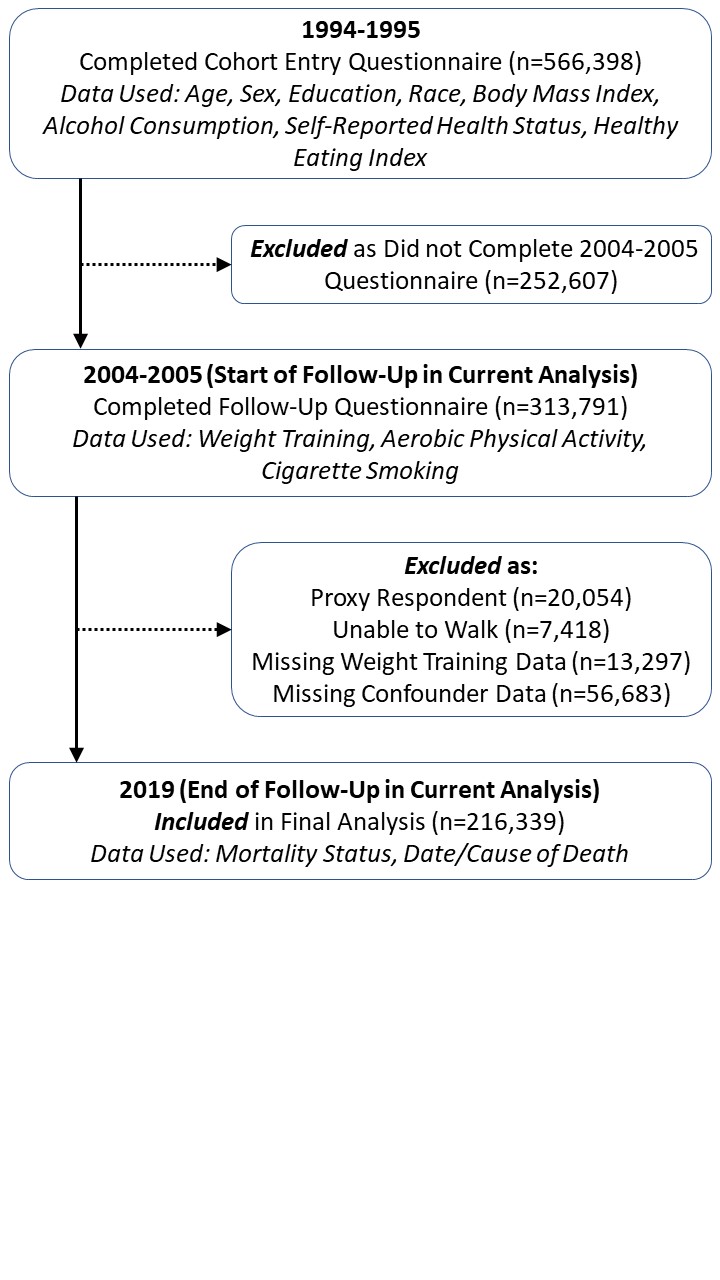

Supplement: dyae074_Supplementary_Data [file dyae074_supplementary_data.docx]
